# Rescue Stenting for Refractory Large Vessel Occlusions in the Thrombectomy Era: Intracranial Use of Coronary Stents in Low-mid Economic Settings

**DOI:** 10.7759/cureus.23847

**Published:** 2022-04-05

**Authors:** Kalyan Chakravarthy Sajja, Vikram Huded, Chintan Prajapati, Shailesh Male, Mukesh Kumar Sharma, Shirpal Shah, Vikram Bohra, Sudheer Chakravarthi, Lakshmi Sudha Prasanna, Pradeep Reddy Sura, Srinivasan Paramasivam, Vamsi Krishna Gorijala, Anusha Guntamukkala, Kumarvelu Somasundaram, Rama Tharaknath Vemuri

**Affiliations:** 1 Endovascular Neurosurgery, Thomas Jefferson University, Willow Grove, USA; 2 Neuroscience, Narayana Health City Hospital, Bangalore, IND; 3 Neurology, Narayana Health City Hospital, Bangalore, IND; 4 Neurology, Vidant Medical Center, Greenville, USA; 5 Neurology, Apollo Hospitals, Ahmedabad, IND; 6 Neurology, Arihant Neurocenter, Nasik, IND; 7 Neurology, Rukmani Birla Hospital, Jaipur, IND; 8 Neurology, Latha Institute of Neurosciences, Vijayawada, IND; 9 Neurology, Apollo Hospitals, Hyderabad, IND; 10 Neurology, Life Neuro Vascular Institute, Guntur, IND; 11 Neurosurgery, Apollo Hospitals, Chennai, IND; 12 Neurology, Guntur Medical College, Guntur, IND; 13 Neurology, Ramesh Hospitals, Guntur, IND

**Keywords:** coronary stenting, failed mechanical thrombectomy, rescue stenting, large vessel occlusion, mechanical thrombectomy

## Abstract

Background: Failed mechanical thrombectomy due to a refractory emergent large vessel occlusion (RELVO) in patients presenting with an acute stroke poses a major challenge to the outcomes.

Objective: We demonstrate the use of coronary stents in the intracranial circulation as rescue stenting for an already expensive mechanical thrombectomy procedure in a mid-low socioeconomic setting.

Methods: A retrospective, multicenter study was conducted between December 2015 and January 2021. The studied cohort were patients who required the use of a rescue stenting using a coronary stent for emergent large vessel occlusion to avoid failed recanalization. Failed recanalization was defined as failed vessel recanalization after at least two passes. Patient demographic data, procedure specifics, type of stent used, and procedural outcomes were collected.

Results: A total of 26 patients with acute ischemic stroke were included from eight different centers across India. Out of 26 patients, 19 (73.0%) were male and seven were female (26.9%). The mean age was 53.6 years, the youngest patient was 23 years old and the eldest was 68 years old. Seven patients (26.9%) had posterior circulation stroke due to occlusion of the vertebral or basilar artery and 19 patients (73.0%) had anterior circulation stroke median NIHSS at presentation was 16 (range 10 to 28) in anterior circulation stroke and 24 (range 16 to 30) in posterior circulation stroke. Intravenous thrombolysis with tissue plasminogen activator (IV tPA) was given in three patients (11.5%). The hospital course of two patients was complicated by symptomatic intracranial hemorrhage (sICH), which was fatal. Favorable revascularization outcome and favorable functional outcome was achieved in 22 patients (84.6%), three patients passed away (11.5%), and one patient was lost to follow up.

Conclusions: Overall, our study finds that rescue stenting using coronary stents can potentially improve outcomes in refractory large vessel occlusions while minimizing costs in low-mid economic settings.

## Introduction

Intracranial stenting was employed for stroke caused by large vessel occlusions in the pre-thrombectomy era. Mechanical thrombectomy (MT) has since emerged as the standard of care for emergent large vessel occlusion (ELVO) after multiple randomized controlled trials showed the benefits of recanalization with stent retrievers. The common technique for MT is deploying a retrievable stent across the occlusion followed by retrieving it into an aspiration catheter [1]. The precursors of these stent retrievers were permanently deployable stents designed for stent-assisted aneurysm treatment. As MT techniques evolved, these devices were modified into the retrievable devices that are being used today. Retrieval of the stent avoids complications associated with permanent stent implantation like thrombo-embolic phenomenon, antiplatelet therapy risks and failures, in-stent thrombosis, and in-stent stenosis. Despite these significant advancements, recanalization is not achieved in about 12%-29% of the mechanical thrombectomies [2-4]. ELVOs that are “refractory” to the standard techniques (RELVO) remain a challenge and are thought to be from etiologies such as intracranial atherosclerotic disease (ICAD), recurrent in situ thrombosis, or iatrogenic vessel injury [4-6]. Some experts perform intracranial stenting to bailout, a technique that was common before the modern thrombectomy era [7,8]. The intracranial stents are self-expanding, low profile, and deployed through the microcatheter. However, their cost is a limiting factor in developing countries like India. We demonstrate our multicenter experience of utilizing balloon-mounted stents in intracranial circulation, a less expensive alternative to self-expanding stents.

## Materials and methods

Patient population

In a retrospective design, data were collected from eight centers across India between December 2015 and January 2021. All eight institutes in our study had a collective IRB named “Narayana Health Academic Ethics Committee.” The committee reviewed our study and approved it since there are no barriers, either scientifically or ethically, to the conduct of the study as it is retrospective and involved no risk. The patients with failed recanalization with standard attempts of MT, who underwent treatment with balloon mounted stent were identified. Patient demographics, procedure specifics, and clinical outcomes were collected. 

Procedural technique

All the procedures performed on the patients were in accordance with the standardized guidelines and regulations. They were performed under conscious sedation or general anesthesia at the discretion of the operator.

Using a 180 cm 0.014 wire, balloon mounted stent is tracked into the intracranial circulation. The intermediate catheters that are typically used for stroke are also not compatible lengthwise with the coronary stent/balloon systems. To avoid using a new intermediate catheter, the coronary stents can be tracked over a wire via any standard 0.088 inner diameter guide catheter or a shorter intermediate catheter. Since it is an ELVO, operators used the stents that were readily available to them. Some operators used bare metal stents while others used drug-eluting stents. Another area of difficult decision-making is with the antiplatelets. Different operators used different regimens of antiplatelets. In general, the goal is to load the patient with dual antiplatelet and to start on a continuous infusion of IV tirofiban with or without a bolus while the oral antiplatelet medications reach a therapeutic target. For patients who received IV tPA, some of the operators used IV tirofiban as a single bolus dose without the infusion to avoid intracranial bleeding complications. Many of the operators also started preferring Ticagrelor as opposed to Clopidogrel as the second antiplatelet agent in addition to aspirin.

Study endpoints

The primary outcome was a favorable functional outcome, defined as a modified Rankin score of 0-2 at 90 days. Secondary outcomes were symptomatic intracranial hemorrhage (sICH), defined as an increase in NIHSS score by at least four points, and favorable modified thrombolysis in cerebral ischemia (mTICI) defined as 2b-3.

## Results

A total of 26 patients with acute ischemic stroke with RELVO were identified from eight centers across India. We believe the etiology was mostly atherosclerosis. There were no patients with iatrogenic dissection, cardiogenic stroke, vasospasm , etc. Out of 26 patients, 19 (73.0%) were male and seven were female (26.9%). The mean age was 53.6 years, the youngest patient was 23 years old and the eldest was 68 years old. Seven patients (26.9%) had posterior circulation stroke due to occlusion of the vertebral or basilar artery and 19 patients (73.0%) had anterior circulation stroke due to occlusion of either intracranial internal carotid artery (six patients) or proximal middle cerebral artery (12 patients). Among patients with anterior circulation stroke (19), 13 patients (68.4%) had left hemispheric, and eight patients (42.1%) had right hemispheric involvement. median NIHSS at presentation was 16 (range 10 to 28) in anterior circulation stroke and 24 (range 16 to 30) in posterior circulation stroke. Intravenous thrombolysis with tissue plasminogen activator (IV tPA) was given in three patients (11.5%). Dual antiplatelet, aspirin combined with Ticagrelor or Clopidogrel were used in all cases. Intravenous glycoprotein IIb/IIIa inhibitors (abciximab or tirofiban) were used in eight patients (30.7%). The stents were used at the discretion of the operator. Since it is an ELVO, operators used the stents that were readily available to them. Xience Xpedition (Abbot, Plymouth, MN, USA) and Xience V (Abbot, Plymouth, MN, USA) were the most commonly used coronary stents. A favorable mTICI score was achieved in 22 patients (84.6%) (Table 1). The Hospital course of two patients was complicated by sICH which was fatal. No perforations or dissections were noted. Median NIHSS at discharge was 4 (range 0 to 20) in patients with anterior circulation stroke (excluding the two patients that suffered ansICH). Among patients with posterior circulation occlusion, the median NIHSS for the five patients was 4.5 (range 0 to 12). Two patients had expired and one did not have documented NIHSS at discharge. Favorable functional outcome was achieved in 22 patients (84.6%), three patients expired (11.5%), and one patient was lost to follow up (Table 1).

**Table 1:** Patient details for intracranial rescue stenting mTICI : Modified Thrombolysis in Cerebral Infarction Scale, NIHSS: National Institutes of Health Stroke Scale, mRS: modified Rankin Scale, ICH: Intracranial hemorrhage, tPA: Tissue plasminogen activator, MCA: Middle Cerebral Artery, ICA: Internal Carotid Artery, M1: M1 segment (sphenoidal/horizontal segment) of MCA, V4: V4 segment (intradural/intracranial part) of Vertebral Artery

Patient	Age	Sex	Location of stroke	Passes	mTICI Score	Pre-op NIHSS	Discharge NIHSS	3-month mRS	sICH	Intravenous tPA	Anti-platelet drugs used	Stent used
Patient 1	33	M	Left M1	3	2b	14	6	0	no	yes	Aspirin + Ticagrelor	Promus Elite 3 mm x 15 mm
Patient 2	58	M	Left M1	3	2b	28	8	1	no	yes	Aspirin + Ticagrelor	Promus Elite 3 mm x 21 mm
Patient 3	42	M	Left ICA	2	3	22	0	0	no	No	Aspirin + Clopidogrel	Xience Xpedition 3.25 mm x 15 mm
Patient 4	62	F	Left supraclinoid ICA	3	3	16	4	1	no	No	Aspirin + Clopidogrel	Ultimaster 3.5 mm x 15 mm
Patient 5	58	M	Right MCA	2	2b	12	10	2	no	No	Aspirin + Ticagrelor	Ultimaster 2.5 mm x 12 mm
Patient 6	38	M	Right MCA	3	3	14	2	1	no	No	Aspirin + Ticagrelor	Xience Xpedition 2.75 mm x 15 mm
Patient 7	55	M	Left Vertebral artery	4	2b	24	12	2	no	No	Aspirin + Clopidogrel	Ultimaster 3.75 mm x 18 mm
Patient 8	65	M	Left Vertebral artery	3	3	16	7	1	no	No	Aspirin + Clopidogrel	Xience Xpedition 4 mm x 18 mm
Patient 9	67	M	Basilar artery	2	3	30	0	0	no	No	Aspirin + Ticagrelor	Xience Xpedition
Patient 10	48	M	Left MCA	2	3	22	2	0	no	No	Aspirin + Ticagrelor	Promus Elite 2.25 mm x 12 mm
Patient 11	55	M	Left MCA	3	3	20	20	2	no	No	Aspirin + Ticagrelor	Xience Xpedition 2.5 mm x 12 mm
Patient 12	67	F	Left Petro-Cavernous junction ICA	2	3	18	4	0	no	no	Tirofiban, Aspirin + Ticagrelor	Xience Xpedition 2.5 mm x 15 mm
Patient 13	40	M	Left MCA	2	3	10	4	0	no	no	Aspirin + Ticagrelor	Xience V 2.75 x 15 mm
Patient 14	58	M	Left MCA	2	3	17	0	0	no	no	Tirofiban	Resolute Onyx 2.5 x 12 mm
Patient 15	65	M	Basilar Artery	2	2b	26	2	0	no	no	Aspirin + Ticagrelor	Resolute Onyx 2.5 mm x 12 mm
Patient 16	60	M	Left Cavernous ICA	2	3	16	4	0	no	no	NA	Bare Metal 2.75 mm x 12 mm
Patient 17	57	M	Right ICA	4	3	16	NA	0	no	no	Abciximab	Bare Metal 2.75 mm x 12 mm
Patient 18	26	F	Left MCA	4	3	18	0	0	no	no	Abciximab	Xience V 2.75 mm x 12 mm
Patient 19	23	F	Right MCA	4	3	18	0	0	no	no	Abciximab	Bare Metal 2.75 mm x 12 mm
Patient 20	64	F	Basilar Artery	2	0	29	Death	6	yes	no	NA	Liberte 2.75 mm x 12 mm
Patient 21	68	M	Mid- Basilar Artery	2	0	16	Death	6	yes	no	Tirofiban + Aspirin	Xience V 2.75 mm x 12 mm
Patient 22	56	M	Left Cavernous ICA	2	3	12	0	0	no	no	NA	Xience V 3 mm x 15 mm
Patient 23	43	M	Left V4, Basilar Artery	2	0	20	Death	6	no	yes	Tirofiban + Aspirin + Clopidogrel	Xience V 3.25 mm x 23 mm
Patient 24	68	F	Left MCA	2	3	26	15	NA	no	no	Aspirin + Clopidogrel	Xience V 2.5 mm x 12 mm
Patient 25	63	F	Right Cavernous ICA	2	3	10	6	1	no	no	Aspirin + Clopidogrel	Xience Prime 4 mm x 15 mm
Patient 26	54	M	Right MCA	3	3	13	4	1	no	no	Tirofiban	Xience Xpedition 3 mm x 8 mm

## Discussion

The goal of recanalization is to restore the arrested blood flow in the affected vascular territory and to salvage the ischemic brain tissue that is at risk of permanent damage. The main predictor of a good clinical outcome is successful recanalization which is indicated by complete or greater than 50% reperfusion of the distal branches in the expected territory (TICI 2b/3). The recently developed thrombectomy techniques that combine stent retrieval with aspiration via intermediate large-bore catheters have significantly increased the rate of successful reperfusion. Despite this, MT is not successful in about 12%-29% of the patients [2-4]. In such cases of RELVOs, recanalization is not achieved despite repeated attempts of MT. The natural history of non-recanalized occlusions is uniformly poor. The etiologies of RELVOs could be a severe underlying atherosclerotic disease with in situ thrombo-occlusions, intra-arterial dissections, or iatrogenic vasospasms [4]. Given the time-sensitive nature of successful clinical outcomes, the early recognition of RELVOs and the adoption of an alternative approach to treatment could be crucial factors for a successful outcome. There are multiple techniques described as bail-out procedures such as IA tPA, IA antiplatelet infusion, balloon angioplasty, or permanent stenting, with no particular consensus among these various techniques [9,10]. In this study, we presented our multicentric experience of rescue intracranial stenting with balloon-mounted stents as a bailout technique to achieve successful recanalization and better clinical outcomes.

Of all the described techniques, intracranial stenting appears to have better chances of success with improved clinical outcomes. Administration of intra-arterial tPA has been shown to increase the risk of intracranial hemorrhage [11]. Similarly, Kellert et al reported that antiplatelet infusion during endovascular thrombectomy led to higher rates of fatal intracranial hemorrhages [12,13] Recently, some experts report using Y-stent retriever thrombectomy (using two stent retrievers in each of the bifurcation branches and performing thrombectomy) that tends to be a highly complex and possibly higher risk of complications [14]. Balloon angioplasty is a promising technique. However, it can often cause endothelial disruption and has a higher rate of re-occlusion. Permanent stent implantation has been recently advocated as a bailout for failed MT in multiple studies [15,16]. Stent deployment seems to be an attractive and biologically feasible option as it may maintain vessel patency where otherwise there might be a persistent occlusion and a higher likelihood of a poor clinical outcome. The implantation of stents for revascularization of large vessel occlusions in the setting of acute ischemic stroke has been described in some leading studies [15,17-19]. The reports suggest a higher rate of recanalization and a low rate of intracranial hemorrhage with rescue intracranial stenting. A meta-analysis of rescue stenting by Wareham et al. reported a weighted pooled estimate of successful recanalization using rescue stents to be 71% and a favorable clinical outcome being achieved at 43% [8]. Similarly, Chang et al. showed 64.6% successful recanalization after rescue stenting without an increase in sICH and mortality [4].

Self-expanding intracranial stents have been developed to treat ICAD (Wingspan) and wide-neck aneurysms. Experts often turn to these stents for the treatment of RELVOs are the workhorse to treat intracranial atherosclerotic lesions [20-22]. The intracranial self-expanding stents are flexible and exert less radial outward force during deployment thus increasing the deliverability and safety [23]. They have superior navigability with fewer risk of vasospasm and side branch occlusion (snow plowing effect) [24]. Further, they can be easily deployed in adjacent vessels of a different caliber. In the SARIS trial, mid-term angiographic follow-up data demonstrated that none of the survivors developed an in-stent stenosis [25,26]. However, these stents are expensive costing almost >10 times those of drug-eluting balloon mounted stents and >15 times of bare metal balloon mounted stents in developing countries like India (Table 2). Given limited resources, it is not uncommon for practitioners to use balloon-mounted stents for the treatment of intracranial lesions. These stents are however not encouraged due to concerns of high risk of restenosis, intimal proliferation, thrombus displacement, and risk of vessel injury. In addition to affordability, these stents provide a simple delivery platform and single-pass deployment without the need for length exchanges for angioplasty and stenting. However, there are some patients with a TICI score of 0. We do not know why the stent failed to remain open and recanalization was not achieved. We presume it could be due to an insufficient loading dose of anti-platelets or it could be other factors.

**Table 2:** Price comparison of common stents used in India

Stent	Maximum Retail Price (in rupees)	Price (in US dollars)
INTRACRANIAL STENTS		
Neuroform Atlas (Stryker)	285,000	3,769.43
Enterprise (Codman)	290,000	3,835.56
Credo (Acandis)	350,000	4,629.12
Wingspan (Stryker)	180,000	2,380.69
CORONARY STENTS		
Xience Xpedition (Abbott)	32,351	427.88
Xience Prime (Abbott)	24,999	330.64
Xience V(Abbott)	32,351	427.88
Resolute Onyx (Medtronic)	32,351	427.88
Synergy (Boston scientific)	32,351	427.88
Liberte (Boston scientific)	8,320	110.04
Promus Elite (Boston scientific)	32,351	427.88
Ultimaster (Terumo)	32,351	427.88
Bare Metal stent	7,260	96.02

Our study suggests that the use of balloon-mounted stents as a bailout technique for the treatment of RELVOs appears to be safe and effective. Study limitations include the retrospective nature of the study, variability in practice from access to provider experience, and the absence of a comparison group. Nevertheless, this is probably the largest case series to this date on the use of balloon-mounted stents as a rescue bailout technique for RELVOs.

## Conclusions

Our experience finds that rescue stenting to achieve recanalization can potentially improve outcomes in RELVOs. Using coronary stents in this regard can be a cost-effective solution. Coronary stents are readily available and are several times cheaper compared to the self-expandable stents that are used in intracranial circulation. However, large randomized studies with long-term follow-up and cost-benefit analysis have to be performed to evaluate if achieving recanalization in large vessel occlusions refractory to standard thrombectomy techniques with rescue coronary stenting would be helpful and economically superior.

The operators in our series used the most readily available coronary stent at their disposal to achieve recanalization without a distinction between bare-metal coronary stents and drug-eluting coronary stents. This was likely due to the emergent nature of the RELVO. Larger comparative studies and long-term data between bare-metal stents and drug-eluting stents in the intracranial circulation are also lacking. Ultimately, achieving recanalization results in a smaller stroke volume as opposed to non-recanalization. Non-recanalization leaves the emergent large vessel occlusion to its natural history of significant disability and mortality. Achieving successful recanalization should be the goal of this effective intervention for disabling strokes.
